# Ultrasonic Elastography of the Rectus Femoris, a Potential Tool to Predict Sarcopenia in Patients With Chronic Obstructive Pulmonary Disease

**DOI:** 10.3389/fphys.2021.783421

**Published:** 2022-01-05

**Authors:** Mingming Deng, Xiaoming Zhou, Yanxia Li, Yan Yin, Chaonan Liang, Qin Zhang, Jingwen Lu, Mengchan Wang, Yu Wang, Yue Sun, Ruixia Li, Liming Yan, Qiuyue Wang, Gang Hou

**Affiliations:** ^1^Department of Pulmonary and Critical Care Medicine, Center of Respiratory Medicine, China-Japan Friendship Hospital, Beijing, China; ^2^Graduate School of Peking Union Medical College, Chinese Academy of Medical Sciences, Peking Union Medical College, Beijing, China; ^3^National Center for Respiratory Medicine, Beijing, China; ^4^Institute of Respiratory Medicine, Chinese Academy of Medical Sciences, Beijing, China; ^5^National Clinical Research Center for Respiratory Diseases, Beijing, China; ^6^Department of Pulmonary and Critical Care Medicine, Fourth Hospital of China Medical University, Shenyang, China; ^7^Respiratory Department, The First Affiliated Hospital of Dalian Medical University, Dalian, China; ^8^Department of Pulmonary and Critical Care Medicine, First Hospital of China Medical University, Shenyang, China; ^9^Department of Pulmonary and Critical Care Medicine, China-Japan Friendship Hospital Affiliated to Capital Medical University Beijing, Beijing, China; ^10^Peking University China-Japan Friendship School of Clinical Medicine, Beijing, China

**Keywords:** ultrasonic elastography, skeletal muscle dysfunction, sarcopenia, COPD, rectus femoris

## Abstract

**Purpose:** Skeletal muscle dysfunction is common in patients with chronic obstructive pulmonary disease (COPD) and is associated with a poor prognosis. Abnormal muscle quantity of the lower limbs is a manifestation of skeletal muscle dysfunction in patients with COPD. Shear wave ultrasound elastography (SWE) is a novel and possible tool to evaluate qualitative muscle parameters. This study explores the feasibility of SWE to measure the stiffness of the rectus femoris and evaluates its value in predicting sarcopenia in patients with COPD.

**Methods:** Ultrasound examination of the rectus femoris was performed to determine the mean elasticity index (SWE_mean_), cross-sectional area (RF_csa_), and thickness (RF_thick_) using grayscale ultrasonography (US) and SWE in 53 patients with COPD and 23 age-matched non-COPD healthy controls. The serum levels of circulating biomarkers (GDF15, resistin, and TNF-α) were measured using ELISA. The definition of sarcopenia followed the guidelines from the Asian Working Group for Sarcopenia. Receiver operating characteristic (ROC) curve analysis of the SWE_mean_, RF_thick_, and RF_csa_ was used to evaluate their predictive ability for sarcopenia.

**Results:** The intraobserver and interobserver repeatability of SWE performance was excellent (all correlation coefficients > 0.95; *p* < 0.05). The SWE_mean_ of the rectus femoris in patients with COPD (8.98 ± 3.12 kPa) was decreased compared with that in healthy controls (17.00 ± 5.14 kPa) and decreased with advanced global initiative for chronic obstructive lung disease (GOLD) stage. Furthermore, SWE_mean_ was found to be independent of sex, height, and body mass, and a lower SWE_mean_ in patients with COPD was positively associated with reduced pulmonary function, worse physical function, poor exercise tolerance, decreased muscle strength, and worse dyspnea index score. The correlation between physical function [five-repetition sit-to-stand test (5STST)], muscle function, and SWE_mean_ was higher than those of RF_thick_ and RF_csa_. In addition, SWE_mean_ was negatively correlated with serum GDF15 levels (*r* = −0.472, *p* < 0.001), serum resistin levels (*r* = −0.291, *p* = 0.035), and serum TNF-α levels (*r* = −0.433, *p* = 0.001). Finally, the predictive power of SWE_mean_ [area under the curve (AUC): 0.863] in the diagnosis of sarcopenia was higher than that of RF_thick_ (AUC: 0.802) and RF_csa_ (AUC: 0.816).

**Conclusion:** Compared with grayscale US, SWE was not affected by the patient’s height, weight, or BMI and better represented skeletal muscle function and physical function. Furthermore, SWE is a promising potential tool to predict sarcopenia in patients with COPD.

## Introduction

Chronic obstructive pulmonary disease (COPD) is a chronic airway disease characterized by persistent respiratory symptoms and airflow limitation (Riley and Sciurba, 2019). It is often accompanied by multiple complications (Vanfleteren et al., 2016), including those associated with the cardiovascular system, digestive system, blood, musculoskeletal system, etc. Skeletal muscle dysfunction, which is present in 1/3 of patients with COPD, is related to poor prognosis, increased hospitalization rate and mortality (Maddocks et al., 2014). Patients with skeletal muscle dysfunction have an increased dyspnea index score [modified British medical research council (mMRC) score], decreased exercise tolerance, decreased exercise capacity, and reduced activity (Degens et al., 2015; Abdulai et al., 2018). Early identification and intervention of skeletal muscle dysfunction is important for improving the quality of life of patients and improving the prognosis of the disease.

Sarcopenia is one of the important systemic symptoms of patients with COPD (Sepúlveda-Loyola et al., 2020). The diagnostic process for sarcopenia requires specific equipment (dual-energy X-ray or CT) and professional training (Cruz-Jentoft et al., 2019) and is inconvenient to conduct in developing countries and primary medical institutions. The diagnosis of sarcopenia involves a combined assessment of muscle quantity and quality (muscle strength or physical performance). Ultrasonography (US) is a user-friendly noninvasive tool that is nonradioactive and has been proven to measure muscle quantity (Seymour et al., 2009). Several studies have shown that the size of the rectus femoris based on grayscale ultrasound measurements was decreased in patients with COPD compared with that in healthy controls and was related to pulmonary function, fat-free mass, and physical performance (Seymour et al., 2009; Ye et al., 2017). However, grayscale ultrasonography only provides a quantification of the amount of muscle and is unable to determine muscle quality and function.

Shear wave ultrasound elastography (SWE) is a novel ultrasound technology that can provide a direct and real-time quantification of the mechanical properties of tissues and additional information about muscle quality (Creze et al., 2018). SWE determines the stiffness of the tissue by measuring the elasticity index based on the degree of distortion under the application of an external force (shear waves; Sigrist et al., 2017) and is valuable for diagnosing breast (Youk et al., 2017), thyroid (Magri et al., 2016), and liver diseases (Conti et al., 2016). Our previous study (Jiang et al., 2019) applied SWE for the first time to diagnose malignant pleural effusion, suggesting its clinical application potential in respiratory diseases. Recently, Xu et al. (2021) suggested that SWE may be employed as an effective tool for the quantitative evaluation of diaphragm stiffness in patients with stable COPD. However, the clinical value of SWE in evaluating lower limb muscle and predicting sarcopenia in patients with COPD is unclear.

In this prospective study, we first explored the reliability of ultrasound SWE to evaluate the rectus femoris and the correlation between the mean elasticity index (SWE_mean_) and clinical parameters. Additionally, the relationship between the measurement of the rectus femoris based on US or SWE and muscle quality and function was compared. Finally, we explored the potential value of SWE in the prediction of sarcopenia in patients with COPD.

## Materials and Methods

### Study Design and Patients

From December 2019 and December 2020, a total of 53 patients with COPD (≥40 years old) and 23 age-matched healthy controls from the First Hospital of China Medical University (Shenyang, China), the Fourth Hospital of China Medical University (Shenyang, China), and the First Hospital of Dalian Medical University (Dalian, China) were recruited for this prospective observational study. The inclusion criterion was a diagnosis of stable COPD according to the Global Initiative for Chronic Obstructive Lung Disease (GOLD) criteria. The exclusion criteria were as follows: COPD exacerbation within the last 1 month; presence of severe cardiovascular disease or active lung disease; concomitant disease affecting the musculoskeletal system; long-term systemic steroid therapy; and inability to read or understand the informed consent documents. Clinical features, including age, sex, height, and weight, were obtained from medical records. The study was approved by the research ethics committees of the First Hospital of China Medical University (No. 2019-144-2), and written informed consent was obtained from all patients.

### Pulmonary Function and Assessment of Modified British Medical Research Council Scale and COPD Assessment Test

Spirometry measurements (pre-bronchodilator) were performed following the American Thoracic Society and the European Respiratory Society guidelines using a Jaeger MasterScreen system (Jaeger, Viasys Healthcare GmbH, Hoechberg, Germany). Dyspnea symptoms were measured using the Chinese version of the modified British medical research council (mMRC) dyspnea scale (Bestall et al., 1999; Cui et al., 2017), and health status was measured using the Chinese version of the COPD assessment test (CAT; Jones et al., 2009; Zhou et al., 2013).

### Quadriceps Muscle Strength

Quadriceps muscle strength (QMS) was measured *via* a dynamometer (type: microFET2™; Hoggan, Salt Lake City, UT, United States) following the instructions in the manufacturer’s manual and previous studies (O’Shea et al., 2007; Zhang et al., 2018). The patients’ knee was flexed to 90°, and the dynamometer plate was placed. The anterior end of the dynamometer was located 5 cm proximal to the lateral malleolus on the anterior surface of the leg and perpendicular to the long axis of the tibia. The participant was then instructed to generate a maximal knee extension force to hold the line in the same position for a duration of 4 s by pushing against the dynamometer plate to which the investigator applied increasing force with encouragement. The same steps were repeated twice, with an intervening interval of 30–60 s. The average value of the last two assessments for each lower limb was recorded as the maximum unilateral contraction force. Then, the average contraction force on both sides was used to obtain the QMS.

### Fat-Free Mass Index

The participant’s body fat rate (BFR) was measured by a bioelectrical impedance meter (InBody770; InBody, Seoul, Korea). The fat-free mass index (FFMI) was calculated as follows: FFMI = weight (kg) × (1-BFR)/height (m)^2^.

### Five-Repetition Sit-to-Stand Test

The participant was seated on a chair that measured 48 cm high and had no armrests, with their feet on the ground, back supported by the back of the chair, and hands folded in front of their chest. After hearing the test start command, the participant was asked to stand up and sit down five times as quickly as possible; the time needed to complete the five repetitions was recorded. The participant was given verbal encouragement during the test. The test was performed three times, with 1-min rest intervals. The average of three tests was recorded as the result.

### 6MWT

According to the 2002 American Thoracic Society (ATS) guidelines (ATS Committee on Proficiency Standards for Clinical Pulmonary Function Laboratories, 2002), a closed, long, and straight 30-m indoor corridor was selected. The test method was explained to the patient before the test, and the patient was told to walk as much as possible. If they felt short of breath or experienced chest pain or dizziness, they were allowed to slow down or to stop to rest. If the above symptoms worsened and were not relieved after rest, the test was stopped immediately, and the patient was supervised by the experimenter and encouraged using standardized language. After 6 min, the patient heard the experimenter say “time is up,” which was their indication to stop. The test personnel recorded the distance travelled in meters.

### Measurements of the Thickness and Cross-Sectional Area of the Rectus Femoris

Measurement of the rectus femoris thickness and cross-sectional area was performed as in previous studies (Shrikrishna et al., 2012; Maynard-Paquette et al., 2020). Grayscale ultrasound was performed with an Aixplorer ultrasound scanning system (SuperSonic Imagine, Aix-en-Provence, France). Our ultrasonographers received formal training, and with >10 years of experience. The patient did not engage in strenuous exercise for 72 h, rested quietly for 15 min and then laid on their back on the operating bed, relaxing all their muscles. The researchers set up a bracket to fix the ultrasound probe in place, thereby reducing muscle deformation due to external forces, and placed the ultrasound probe perpendicular to the patient’s dominant leg. The scanning depth was set such that the femur could be detected for orientation. Gentle contraction-relaxation manoeuvres were employed to delineate the muscle septa prior to image acquisition. RF_thick_ and RF_csa_ were calculated after the inner echogenic line of the rectus femoris was outlined by a movable cursor on a frozen image. RF_thick_ and RF_csa_ were recorded as the averages of three consecutive measurements within 10%.

### SWE Measurements

**Figure 1:**
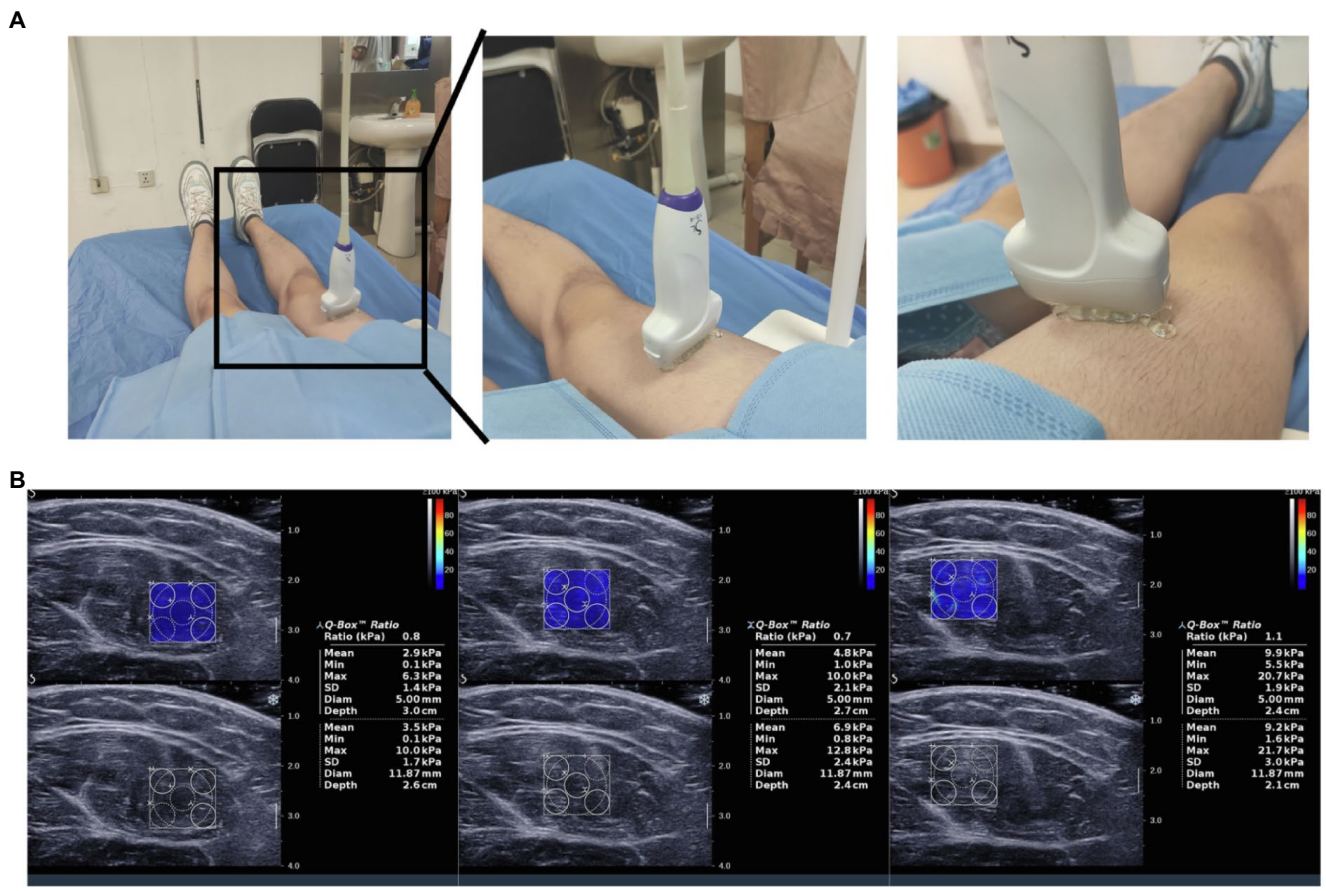
Measurements of rectus femoris stiffness using shear wave elastography. **(A)** The process of shear wave ultrasound elastography assessment; **(B)** Shear wave ultrasound elastography (SWE) images of the rectus femoris are displayed together with the grayscale ultrasound images. After placing a box (frame) over the rectus femoris, a colored image appears, revealing blue and red areas on an elastogram. Dark blue areas correspond to soft tissues, whereas red areas correspond to stiff tissues.

Shear wave elastography was performed using an Aixplorer ultrasound system (Supersonic Imagine, Aix-en-Provence, France) with ShearWave™ Elastography coupled with a 4–15 MHz linear transducer, as previously described in detail (Bercoff et al., 2004; Tanter et al., 2008; Andonian et al., 2016). Grayscale ultrasound was used to identify anatomical structures and to determine the location of the rectus femoris. Next, the user was positioned such that a fixed-size square region of interest (ROI) delimiting the elastographic field of view (SWE box), i.e., a ROI, where shear-wave propagation was analyzed within the muscle, was visible. Three successive SWE acquisitions were performed for the rectus femoris with the transducer in a fixed position (Figure 1B). Images were transferred to an OsiriX workstation (Pixmeo, Geneva, Switzerland) for analysis using a dedicated analysis plugin (QBox, 1.0, Supersonic Imaging). To avoid artifacts in the circular ROIs, five ROIs (5-mm diameter) were placed within a given square SWE color map (Figure 1B). As three SWE acquisitions were performed in succession for each rectus femoris, 15 stiffness measurements were available at each measurement time point for each subject. Same two observers making all SWE measurements in different site.

### Assessment of Sarcopenia

The definition of sarcopenia followed the guidelines from the AWGS (Chen et al., 2020): low muscle mass [bioelectrical impedance (M: <7.0 kg/m^2^, F: <5.7 kg/m^2^)] and low muscle strength [handgrip strength (M: <28 kg, F: <18 kg)] and/or poor physical performance (five-time chair stand test: ≥12 s). Muscle mass was evaluated using bioelectrical impedance analysis (BIA; InBody770; InBody, Seoul, Korea). Muscle strength was assessed by handgrip strength using a JAMAR®Plus^+^ hand dynamometer. The five-time chair stand test was used to assess physical performance.

### Measurement of Circulating Biomarkers

For analysis of circulating biomarkers, plasma sample from patients with COPD were assayed using ELISA kits. TNF-α were assessed by high-sensitivity ELISA kits (#HSTA00E, R&D, Minnesota, United States) following manufacturer’s instructions. And, the GDF-15 and resistin level was determined *via* human GDF-15 quantizing ELISA Kit (#DGD150, R&D, Minnesota, United States) and human resistin quantizing ELISA Kit (#DRSN00, R&D, Minnesota, United States) according to the manufacturer’s instructions.

### Statistical Analyses

Statistical analyses were performed using SPSS 13.0 software (IBM, Armonk, NY, United States). Interobserver and intraobserver reliability were calculated using a two-way random model based on absolute agreement with an intraclass correlation coefficient (ICC) test. The ICCs were interpreted according to the following published guidelines (Shrout and Fleiss, 1979): less than 0.40, poor reliability; 0.40–0.75, fair-to-good reliability; and greater than 0.75, excellent reliability. Continuous variables are expressed as the median values and IQRs, as well as the minimum and maximum values. The differences in the SWE_mean_ between healthy controls and patients with COPD were determined using Mann-Whitney U test, and values of *p* < 0.05 were considered statistically significant. Pearson correlation coefficient analysis was used to compare the correlations of the SWE_mean_, RF_thick_, and RF_csa_ with the clinical features of COPD patients. Receiver operating characteristic (ROC) curve analysis and the area under the curve (AUC) were used to determine the ability of SWE_mean_, RF_thick_, and RF_csa_ to predict sarcopenia.

## Results

### Basic Characteristics

A total of 53 COPD patients and 23 age-matched healthy controls were enrolled in the final analysis. The baseline characteristics of the patients are listed in Table 1. There were no statistically significant differences in age, BMI, or sex between COPD patients and healthy controls. Pulmonary function (FEV_1_, FEV_1_%predicted, FVC, and FEV_1_/FVC) was significantly decreased in patients with COPD compared with that in healthy controls.

**Table 1:** Basic characteristics of the study population.

Characteristics	Healthy control (*n* = 23)	COPD (*n* = 53)	*p*-values
Age, years	63.43 ± 7.81	64.02 ± 8.14	0.751
Male/female, *n*	15/8	40/13	0.358
BMI, kg/m^2^	25.24 ± 3.07	25.07 ± 4.24	0.891
FEV_1_, L	3.05 ± 0.55	1.37 ± 0.68	<0.001
FEV_1_% predicted	104.1 ± 10.43	50.88 ± 21.77	<0.001
FVC, L	3.95 ± 0.55	2.52 ± 0.91	<0.001
FEV_1_/FVC	78.91 ± 4.73	52.17 ± 11.07	<0.001
GOLD A/B/C/D, *n*		8/15/18/12	

BMI, body mass index; FEV_1_, forced expiratory volume in 1 s; FEV_1_% pred, FEV percentage predicted; FVC, forced vital capacity; and GOLD, the global initiative for chronic obstructive lung disease.

### Reliability and Repeatability Levels for Measurements by SWE

First, we determined the reliability and repeatability levels for measurements by SWE in the rectus femoris muscle. Rectus femoris muscle measurements were performed by two observers and at three different times and are shown in Table 2. The assessment of the rectus femoris using SWE yielded excellent intraobserver repeatability ICCs (observer 1: ICC: 0.952, *p* < 0.001; observer 2: ICC: 0.985, *p* < 0.001). Importantly, it was found that the interobserver reliability for the rectus femoris muscle was excellent (ICC: 0.984, *p* < 0.001). These results suggest that SWE is a reliable and repeatable technique for measuring rectus femoris muscle stiffness.

**Table 2:** Reliability and repeatability results of the measurements performed by different observers.

Parameter	Observer 1	Observer 2
**Intraobserver**		
ICC (95% CI)	0.952 (0.863–0.983)	0.985 (0.956–0.995)
*p* value	<0.001	<0.001
**Interobserver**		
ICC (95% CI)	0.984 (0.973–0.991)	
*p* value	<0.001	

### SWE_mean_ in Healthy Control and Patients With COPD

**Figure 2:**
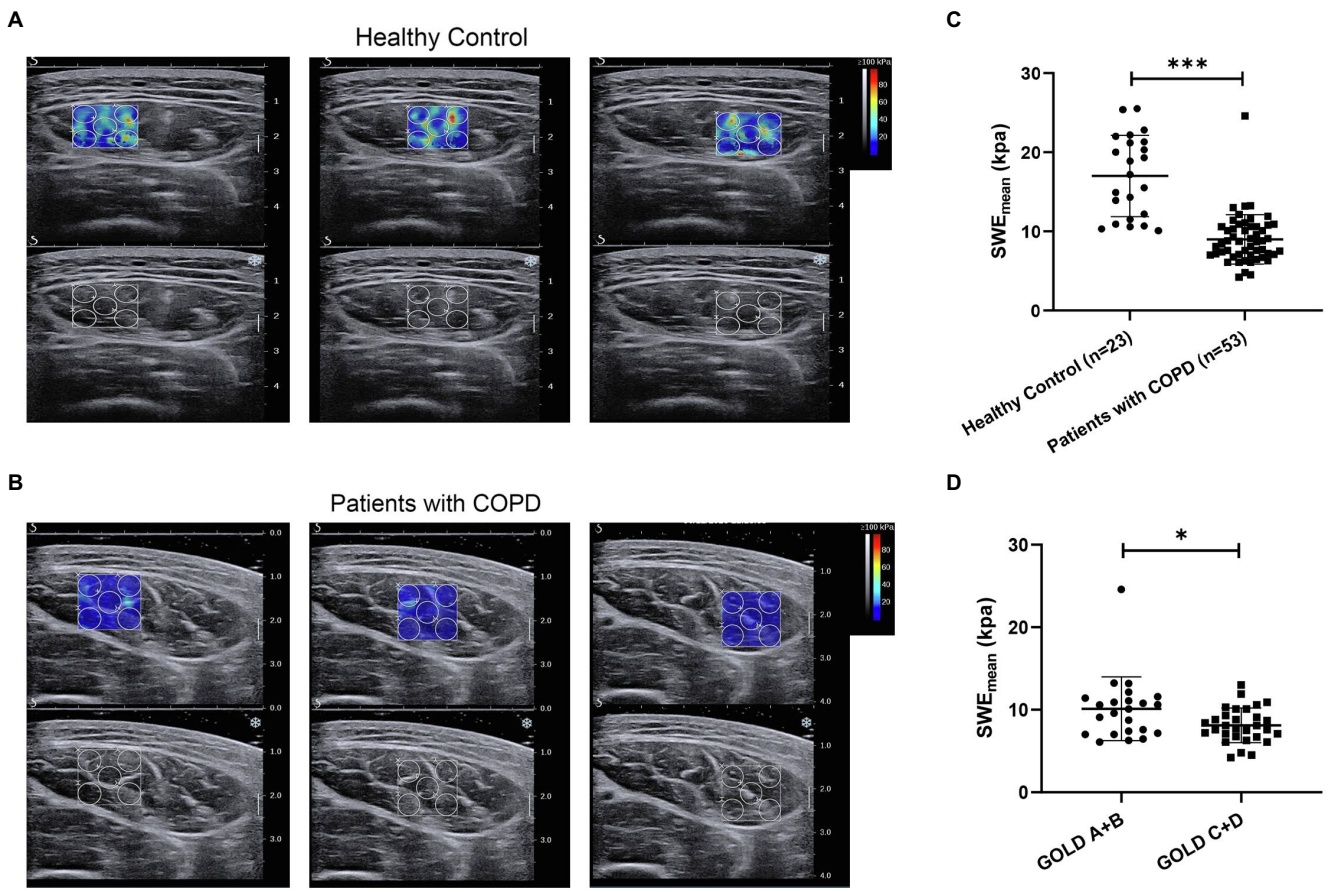
The difference in the mean elasticity indices of rectus femoris ultrasound elastography in patients with chronic obstructive pulmonary disease (COPD) and healthy controls. **(A)** Representative SWE images of the rectus femoris and grayscale ultrasound images in healthy controls; **(B)** Representative SWE images of rectus femoris and grayscale ultrasound images in patients with COPD; **(C)** Difference in mean elasticity index (SWE_mean_) in patients with COPD and healthy controls; and **(D)** Difference in SWE_mean_ in patients with global initiative for chronic obstructive lung disease (GOLD) A and GOLD B and in patients with GOLD C and GOLD D. SWE_mean_: mean SWE elasticity index. *p<0.05; ***p<0.001.

Next, we aimed to assess the clinical value of SWE in patients with COPD. The mean elasticity index (SWE_mean_) of the rectus femoris was measured *via* SWE, and the process is shown in Figures 1A,B. Representative pictures of rectus femoris SWE in healthy controls and patients with COPD are shown in Figures 2A,B. The SWE_mean_ of patients with COPD was significantly lower than that of healthy controls (Figure 2C). In addition, the SWE_mean_ of patients with GOLD stage A and GOLD stage B was significantly higher than that of patients with GOLD stage C and GOLD stage D (Figure 2D). Overall, these results suggest that the rectus femoris stiffness of patients with COPD was decreased compared with that of healthy controls and associated with the severity of the disease.

### Relationships of SWE_mean_ With the Clinical Features of COPD Patients

Next, we explored the relationship between SWE_mean_ and clinical features in patients with COPD. In Table 3, the 6-min walk distance (6MWD; *r* = 0.450, *p* < 0.001), FEV_1_ (*r* = 0.397, *p* = 0.003), FEV_1_%predicted (*r* = 0.317, *p* = 0.021), FEV_1_/FVC (*r* = 0.397, *p* = 0.002), and QMS (*r* = 0.533, *p* < 0.001) were positively correlated with the SWE_mean_. The five-repetition sit-to-stand (5STS; *r* = −0.520, *p* < 0.001), mMRC scores (*r* = −0.301, *p* = 0.029), and CAT scores (*r* = −0.362, *p* = 0.012) were negatively correlated with the SWE_mean_. These results suggest that a lower SWE_mean_ of patients with COPD was positively associated with reduced pulmonary function, worse physical function, poor exercise tolerance, decreased muscle function, and more severe clinical symptoms.

**Table 3:** Correlations among SWE_mean_, RF_thick,_ RF_csa,_ and clinical features.

Variable	SWE		Grayscale ultrasound			
	SWE_mean_, Kpa		RF_thick_, cm		RF_csa_, cm^2^	
	Coefficient	***p***-value	Coefficient	***p***-value	Coefficient	***p***-value
**Demographics**						
Age, years	−0.230	0.098	−0.201	0.148	−0.169	0.228
Height, m	0.174	0.214	0.344	**0.012** ^*^	0.281	**0.041** ^*^
Weight, kg	0.167	0.231	0.299	**0.029** ^*^	0.391	**0.004** ^*^
**Pulmonary function**						
FEV_1_, L	0.397	**0.003** ^*^	0.370	**0.006** ^*^	0.287	**0.037** ^*^
FEV_1_, %predicted	0.317	**0.021** ^*^	0.242	0.081	0.192	0.169
FVC, L	0.266	0.054	0.266	0.071	0.329	**0.016** ^*^
FVC, % predicted	0.189	0.176	0.067	0.635	0.248	0.073
FEV_1_/FVC, %	0.397	**0.003** ^*^	0.401	**0.003** ^*^	0.253	**0.024** ^*^
**Physical function**						
5STS, s	−0.520	**0.039** ^*^	−0.008	0.975	−0.029	0.915
**Exercise tolerance**						
6MWD, m	0.450	**<0.001** ^*^	0.641	**<0.001** ^*^	0.615	**<0.001** ^*^
**Body composition**						
BMI, kg/m^2^	0.130	0.355	0.165	0.237	0.311	0.023
Body fat (%)	−0.038	0.787	−0.099	0.481	0.057	0.682
FFMI (kg/m^2^)	0.354	0.034	0.072	0.675	0.195	0.256
**Muscle function**						
QMS (kg)	0.533	**<0.001** ^*^	0.398	**<0.001** ^*^	0.311	**0.028** ^*^
**Clinical outcome**						
mMRC score	−0.301	**0.029** ^*^	−0.346	**0.004** ^*^	−0.325	**0.012** ^*^
CAT scores	−0.362	**0.012** ^*^	−0.388	**0.006** ^*^	−0.151	**0.031** ^*^

FEV_1_, forced expiratory volume in 1 s; FEV_1_% predicted, FEV percentage predicted; FVC, forced vital capacity; FVC% predicted, FVC percentage predicted; 5STS, five-repetition sit-to-stand test; 6MWD, 6-min walk distance; BMI, body mass index; FFMI, fat-free mass index; QMS, quadriceps muscle strength; mMRC, modified British medical research council; and CAT: COPD assessment test.

* *p* < 0.05.

We compared the relationship between RF_thick_, RF_csa_, and clinical features in patients with COPD. In Table 3, height (*r* = 0.344, *p* = 0.012), weight (*r* = 0.299, *p* = 0.029), 6MWD (*r* = 0.641, *p* < 0.001), FEV_1_ (*r* = 0.370, *p* = 0.006), FEV_1_/FVC (*r* = 0.401, *p* = 0.003), and QMS (*r* = 0.398, *p* < 0.001) were positively correlated with RF_thick_. The mMRC scores (*r* = −0.346, *p* = 0.004) and CAT scores (*r* = −0.388, *p* = 0.006) were negatively correlated with RF_thick_. Height (*r* = 0.281, *p* = 0.041), weight (*r* = 0.391, *p* = 0.004), 6MWD (*r* = 0.615, *p* < 0.001), FEV_1_ (*r* = 0.287, *p* = 0.037), FVC (*r* = 0.329, *p* = 0.016), FEV_1_/FVC (*r* = 0.253, *p* = 0.024), and QMS (*r* = 0.311, *p* = 0.028) were positively correlated with RF_csa_. The mMRC scores (*r* = −0.325, *p* = 0.012) and CAT scores (*r* = −0.151, *p* = 0.031) were negatively correlated with RF_csa_.

### Relationships of SWE_mean_ With the Circulating Biomarkers of COPD Patients

**Figure 3:**
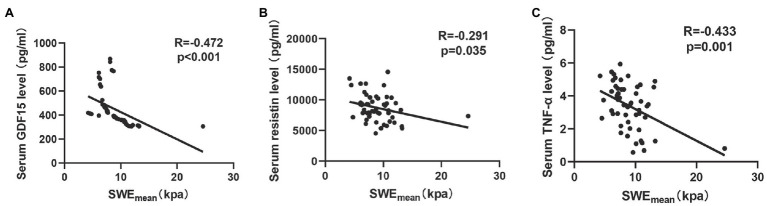
The relationship between circulating biomarkers and the mean elasticity indices of rectus femoris ultrasound elastography in patients with COPD. **(A)** The relationship between SWE_mean_ and serum GDF15 level; **(B)** The relationship between SWE_mean_ and serum resistin level; and **(C)** The relationship between SWE_mean_ and serum TNF-α level. SWE_mean_: mean SWE elasticity index.

Previous studies (Dalle and Koppo, 2020; Looijaard et al., 2021) indicated that circulating biomarkers, including systemic inflammatory markers (Ferrari et al., 2015) and oxidative stress factors (Sepúlveda-Loyola et al., 2021), were associated with muscle quality in COPD. In this study, we analyzed the correlation between SWE_mean_ and a series of circulating biomarkers (GDF15, resistin, and TNF-α). As shown in Figure 3, SWE_mean_ was negatively correlated with serum GDF15 levels (*r* = −0.472, *p* < 0.001), serum resistin levels (*r* = −0.291, *p* = 0.035), and serum TNF-α levels (*r* = −0.433, *p* = 0.001).

### The Potential Value of SWE in the Prediction of Sarcopenia in Patients With COPD

**Figure 4:**
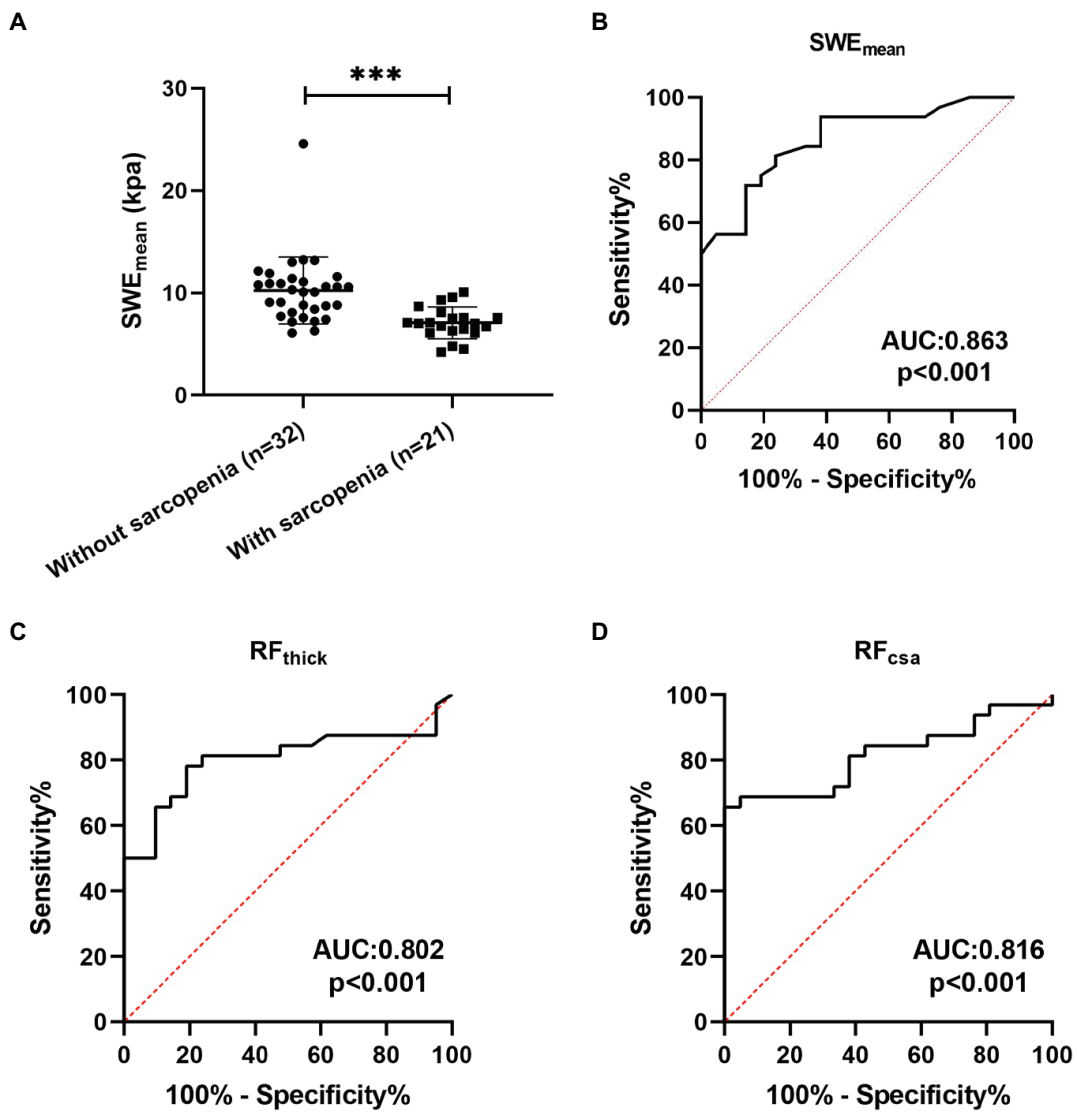
The predictive value of SWE_mean._ **(A)** The SWE_mean_ were differed between patients with sarcopenia and patients without sarcopenia; **(B-D)** Receiver operating characteristic curve analysis of the mean elasticity indices of rectus femoris ultrasound elastography and thickness and cross-sectional area of the rectus femoris for predicting sarcopenia in patients with COPD. SWE_mean_: mean SWE elasticity index; RF_thick_: the thickness of rectus femoris; and RF_csa_: the cross-sectional area of rectus femoris. ***p<0.001.

Sarcopenia is one of the important systemic symptoms in patients with COPD (Sepúlveda-Loyola et al., 2020). Therefore, we explored the potential value of SWE in the diagnosis of sarcopenia in patients with COPD and compared it with grayscale US. Twenty-one (39.62%) patients with COPD had a diagnosis of sarcopenia following the guidelines from the AWGS. As shown in Table 4, pulmonary function (FEV_1_, FEV_1_%predicted, FVC, and FEV_1_/FVC) was significantly decreased in sarcopenic patients with COPD. Patients with sarcopenia showed poor physical function (5STS) and poor exercise tolerance (6MWD). In terms of body composition, FFMI were markedly decreased in sarcopenic patients. The SWE_mean_ of patients with sarcopenia were both significantly lower than those of patients without sarcopenia (Figure 4A). ROC curve analysis was used to explore the ability of SWE_mean_, RF_thick_, and RF_csa_ to predict sarcopenia. As shown in Figure 4, the AUC values for SWE_mean_ (Figure 4B), RF_thick_ (Figure 4C), and RF_csa_ (Figure 4D) were 0.863, 0.802, and 0.816, respectively. These results suggest that the levels of predictive power of SWE_mean_ (from SWE) were higher than those of RF_thick_ and RF_csa_ (from grayscale US).

**Table 4:** Characteristics of the patients with/without sarcopenia.

Variable	Without sarcopenia (*n* = 32)	With sarcopenia (*n* = 21)	*p*-value
**Demographics**			
Age, years	62 ± 7	67 ± 9	**0.002** ^*^
Height, m	1.66 ± 0.07	1.63 ± 0.08	0.317
Weight, kg	71.63 ± 11.92	64.28 ± 17.11	0.074
**Pulmonary function**			
FEV_1_, L	1.57 ± 0.70	1.07 ± 0.55	**0.011** ^*^
FEV_1_, %predicted	55.52 ± 21.21	43.81 ± 21.16	**0.046** ^*^
FVC, L	2.74 ± 0.91	2.19 ± 0.81	**0.028** ^*^
FVC, % predicted	77.69 ± 20.63	69.93 ± 22.11	0.126
FEV_1_/FVC, %	55.34 ± 10.31	47.34 ± 10.65	**0.012** ^*^
**Physical function**			
5STS, s	6.43 ± 1.24	10.37 ± 3.41	**<0.001** ^*^
**Exercise tolerance**			
6MWD, m	416 ± 74.75	318.2 ± 84.71	**<0.001** ^*^
**Body composition**			
BMI, kg/m^2^	25.93 ± 3.77	23.72 ± 4.66	0.051
Body fat (%)	29.34 ± 5.54	30.18 ± 6.88	0.531
FFMI (kg/m^2^)	18.80 ± 2.01	15.79 ± 1.96	**<0.001** ^*^
**Muscle function**			
QMS (kg)	48.45 ± 9.65	38.64 ± 9.87	**0.001** ^*^
**Clinical outcome**			
mMRC score	17.96 ± 5.96	20.76 ± 6.88	0.257
CAT scores	2.07 ± 1.07	2.05 ± 1.24	0.832

FEV_1_, forced expiratory volume in 1 s; FEV_1_% predicted, FEV percentage predicted; FVC, forced vital capacity; FVC% predicted, FVC percentage predicted; 5STS, five-repetition sit-to-stand test; 6MWD, 6-min walk distance; BMI, body mass index; FFMI, fat-free mass index; QMS, quadriceps muscle strength; mMRC, modified British medical research council; and CAT, COPD assessment test.

* *p* < 0.05.

## Discussion

To our knowledge, this is the first study to evaluate the clinical value of rectus femoris SWE in COPD patients. In addition, we demonstrate that SWE_mean_ based on SWE can effectively predict sarcopenia in patients with COPD, and the predictive power is higher than that of RF_thick_ and RF_csa_ based on grayscale US.

Shear wave ultrasound elastography is an emerging ultrasound-based imaging method that has been widely used in studies of musculoskeletal disorders (Taljanovic et al., 2017). However, different results have been reported regarding its reliability (Lacourpaille et al., 2012; Hirayama et al., 2015; Cortez et al., 2016). In addition, most previous studies (Lacourpaille et al., 2012; Taş et al., 2017) focused on evaluating the reliability of SWE in healthy individuals. In this study, the intraobserver reliability (ICC: 0.952–0.985) and interobserver reliability (ICC: 0.984) of SWE were perfect based on analyses of 53 patients with COPD and 23 healthy controls. It was found that reliability values for rectus femoris measurements were higher than many reliability values reported in previous studies (Lacourpaille et al., 2012; Taş et al., 2017). This finding could have resulted from several conditions. First, previous studies use the mean of three or six successive measurements (Lacourpaille et al., 2012; Taş et al., 2017). In this study, the mean of 15 successive measurements was used for analysis. Another cause of the different values could be that rectus femoris muscle stiffness measurements were calculated on the largest measurable area. These results suggested that SWE is a reliable and repeatable technique in evaluating rectus femoris mass changes caused by COPD. Using the mean of a multitude of repetitive measurements performed over a wide area could increase the reliability of the measurements.

Most published studies of SWE were in the fields of limb muscles and tendons in chronic myopathy (Mathevon et al., 2018) and sports medicine (Friede et al., 2020; Taş et al., 2020). Researchers evaluated muscle function by detecting changes in the elasticity index of limb muscles. A lower elasticity index corresponded to lower tissue stiffness. The decrease in muscle stiffness could reflect muscle fiber atrophy (Wen et al., 2018) or a combination of muscle edema, inflammation or lipid accumulation, and fiber atrophy (Alfuraih et al., 2019). In this study, we found that the SWE_mean_ of patients with COPD was decreased compared with that of healthy controls and was associated with the severity of the disease. SWE_mean_ was found to be independent of sex, height, and body mass, and a lower SWE_mean_ of patients with COPD was positively associated with reduced pulmonary function, worse physical function, poor exercise tolerance, decreased muscle function, and more severe clinical symptoms. The correlations between physical function, muscle function, and SWE_mean_ were higher than those of RF_thick_ and RF_csa_ (from grayscale US). This finding could have resulted from several conditions. First, SWE_mean_ was independent of sex, height, and body mass. RF_thick_ and RF_csa_ are affected by individual’s height and weight. Second, residual long-standing lesions in muscle injuries may result in subtle abnormalities that may be hardly visible on grayscale sonography in early periods. Additionally, grayscale US only provides a quantification of the amount of muscle, and biomechanical tissue properties of muscles cannot be assessed. However, SWE could evaluate tissue stiffness and mechanical properties *via* the elasticity index and help to better describe histological changes. Overall, compared with grayscale US, SWE was not affected by the patient’s height, weight, or BMI and better represented skeletal muscle function and physical function.

Sarcopenia has been recognized as a syndrome in various chronic conditions and has complex pathophysiological mechanisms (Rubio-Ruiz et al., 2019; Tournadre et al., 2019). Recent studies indicate that a series of factors underpinning muscle quality, including systemic inflammation, metabolism, and fat infiltration, play an important role in the occurrence and development process of sarcopenia (Dalle and Koppo, 2020; Looijaard et al., 2021). In addition, several studies (Ferrari et al., 2015; Qaisar et al., 2020; Sepúlveda-Loyola et al., 2021) have indicated that circulating biomarkers were associated with muscle quality and could predict sarcopenia in COPD. In this study, we found that SWE_mean_ was negatively correlated with serum GDF15 levels, serum resistin levels, and serum TNF-α levels. Growth differentiation Factor 15 (GDF15) is a member of the transforming growth factor-beta (TGF-β) superfamily (Wang et al., 2021) and a negative regulator of muscle mass and function (Kalinkovich and Livshits, 2015). Previous studies (Patel et al., 2016; Husebø et al., 2017) indicate that GDF15 is associated with muscle mass and promotes muscle wasting and is a predictor of important disease outcomes in patients with COPD. Resistin, a kind of adipokine, plays an important role in skeletal muscle inflammation and skeletal muscle wasting (Nicholson et al., 2018; Mannelli et al., 2020). Systemic inflammation is a common ground for the development and maintenance of sarcopenia (Livshits and Kalinkovich, 2019). TNF-α is a cytokine involved in systemic inflammation (Brenner et al., 2015) and a significant determinant of sarcopenia in patients with stable COPD (Byun et al., 2017). These results further suggest a good correlation between SWE and skeletal muscle function.

Grayscale ultrasound has been used for diagnosing age-related sarcopenia by measuring muscle geometric proportions (Narici et al., 2021). In addition, Chen et al. (2021) reported that SWE could be used for the diagnosis of sarcopenia in patients who have had renal transplants. However, the potential value of grayscale ultrasound and SWE in the diagnosis of sarcopenia in patients with COPD is unclear. In this study, we found that SWE_mean_, RF_thick_, and RF_csa_ could effectively predict sarcopenia in patients with COPD (all AUC > 0.8). The predictive power of SWE_mean_ (AUC: 0.863) was higher than that of RF_thick_ (AUC: 0.802) and RF_csa_ (AUC: 0.816). These results suggest that SWE is a promising tool to identify sarcopenia in patients with COPD.

This study also has several limitations. First, although the patients in our study were from multiple centers, the sample size was relatively small and limited to the northern Chinese population. The clinical application of SWE assessment of the rectus femoris needs further comprehensive and in-depth analysis. Second, although, we determined the reproducibility and reliability of measurements obtained by experienced operators, the reliability of SWE measurements between an experienced operator and the novice operator is still unclear. Therefore, the assessment of the rectus femoris using SWE necessitates training and must be performed by treating physicians or trained radiologists. In addition, the study was limited to stable COPD patients, and whether the findings can be applied to patients with acute exacerbations of COPD and those undergoing pulmonary rehabilitation remains unknown. These data will be provided in a future study.

In conclusion, compared with grayscale US, SWE was not affected by the patient’s height, weight, or BMI and better represented skeletal muscle function and physical function. In addition, SWE is a promising potential tool to predict sarcopenia in patients with COPD.

## Data Availability Statement

The original contributions presented in the study are included in the article/supplementary material, further inquiries can be directed to the corresponding author.

## Ethics Statement

The studies involving human participants were reviewed and approved by the Research Ethics Committee of China Medical University. The patients/participants provided their written informed consent to participate in this study.

## Author Contributions

GH: conceptualization. MD, CL, YY, QZ, JL, MW, YW, and YS: data curation. CL, QZ, JL, MW, YW, and YS: methodology. XZ, YL, and QW: project administration. MD and GH: writing – original draft and writing – review and editing. All authors contributed to the article and approved the submitted version.

## Conflict of Interest

The authors declare that the research was conducted in the absence of any commercial or financial relationships that could be construed as a potential conflict of interest.

## Publisher’s Note

All claims expressed in this article are solely those of the authors and do not necessarily represent those of their affiliated organizations, or those of the publisher, the editors and the reviewers. Any product that may be evaluated in this article, or claim that may be made by its manufacturer, is not guaranteed or endorsed by the publisher.
